# Ranging ecology and resource selection of white‐lipped peccaries (*Tayassu pecari*) in the world's largest tropical agricultural frontier

**DOI:** 10.1002/ece3.10624

**Published:** 2023-10-18

**Authors:** Hugo C. M. Costa, Danielle Storck‐Tonon, Manoel dos Santos‐Filho, Dionei José da Silva, João Vitor Campos‐Silva, Carlos A. Peres

**Affiliations:** ^1^ Programa de Pós‐graduação em Ecologia Instituto Nacional de Pesquisas da Amazonia Manaus Brazil; ^2^ Instituto Juruá Manaus Brazil; ^3^ Programa de Pós‐graduação em Ecologia e Conservação da Biodiversidade Universidade Estadual de Santa Cruz Ilhéus Brazil; ^4^ Programa de Pós‐Graduação em Ambiente e Sistemas de Produção Agrícola Universidade do Estado de Mato Grosso Tangará da Serra Brazil; ^5^ Universidade do Estado de Mato Groso Cáceres Brazil; ^6^ School of Environmental Sciences University of East Anglia Norwich UK

**Keywords:** AKDE, habitat use, home range, telemetry, umbrella species, ungulates

## Abstract

Agricultural commodity production is one the main drivers of deforestation in Legal Brazilian Amazonia resulting in a deforested and/or fragmented landscape formed by forest remnants of different sizes and shape embedded within the agricultural matrix. As an ecosystem engineer and a crucial seed predator, white‐lipped peccaries (*Tayassu pecari*) play a pivotal role in forest structure, biodiversity, and nutrient cycling. However, they are highly sensitive to habitat fragmentation and hunting pressure. White‐lipped peccaries are, therefore, a wide‐ranging “landscape species,” the spatial and ecological requirements of which can be used to guide conservation planning in human‐modified landscapes. Using data from GPS‐tracked individuals in large‐scale mechanized agriculture landscapes in the state of Mato Grosso, Brazil's largest soybean and maize producer, we investiated the home range size and resource selection during both the crop and non‐crop season. We observed a seasonal variation in home range size and an increased selection for croplands during the crop season. White‐lipped peccaries favored native vegetation patches and also exhibited avoidance of locations distant from perennial water bodies and distant cropland locations far from forest remmants. This study can contribute to inform effective conservation strategies and land management practices aimed at preserving suitable habitats and promoting wildlife coexistence with working agricultural landscapes.

## Graphical abstract

White‐lipped peccaries (*Tayassu pecari*) play a crucial role in forest structure, biodiversity maintenance, and nutrient cycling as ecosystem engineers and key seed predators. Using GPS data from tracked individuals in large‐scale mechanized agriculture landscapes in Mato Grosso, the largest producer of soybean and maize in Brazil, we investigate home range size and resource selection during both crop and non‐crop seasons. Our findings revealed a seasonal variation in home range size and a markedly increased selection for croplands during the crop season. The information obtained from this study has significant implications for the development of effective conservation strategies and land management practices aimed at preserving suitable habitats and promoting coexistence between wildlife and human activities.

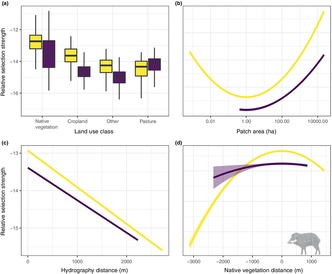

## 1 INTRODUCTION

One‐third of all forests globally were lost to anthropogenic land use, over half of which during the 20th century (FAO, 2020). The Amazon represents over half of the world's remaining tropical forests and provides critical global scale ecosystem services, including climate regulation (Malhi et al., 2008), carbon storage (Avitabile et al., 2016), and biodiversity retention (Pimm et al., 2014). The ~5.2 million km^2^ Legal Brazilian Amazon region includes nine states and spans 61% of the country, but its southern and eastern portions have been rapidly converted into cattle pastures and cropland since the 1970s (Kalamandeen et al., 2018; Montibeller et al., 2020), largely induced by government‐sponsored migration into agrarian settlements (Schneider & Peres, 2015). Commodity production mainly represented by bovine beef and soybean are the main drivers of Amazonian deforestation (Pendrill et al., 2019; Tyukavina et al., 2017), amounting to ~82 Mha of the Legal Brazilian Amazon by late 2022 (MapBiomas 2023). Mato Grosso, the third largest Brazilian state (903,357 km^2^), is the largest beef and soybean producer, accounting for ~32M head of cattle (Instituto Brasileiro de Geografia e Estatística, 2021) and ~11.8 Mha of monoculture cropland during the 2022–2023 harvest season (Compania Nacional de Abastecimento, 2023). Mato Grosso thus hosts the world's largest tropical agricultural frontier, with mechanized cropland expansion – mainly allocated to soybean and maize, resulting in 152,057 km^2^ of cumulative deforestation over the 1988–2022 period (Assis et al., 2019).

Primary native vegetation loss and subsequent fragmentation of the remaining habitat patches reduces carbon storage (Pendrill et al., 2019), alters local climate (Spera et al., 2020), increases wildfires (Alencar et al., 2015), and intensifies the effects of other pervasive but poorly mapped threats such as overhunting (Benítez‐López et al., 2017; Peres, 2001) and human‐wildlife conflicts (Buchholtz et al., 2020; Lima et al., 2019). Although habitat patch area is a key predictor of local species richness in fragmented landscapes (Benchimol & Peres, 2015) matrix permeability and species traits modulate the overall threshold of patch‐scale biodiversity persistence (Bueno & Peres, 2019). Large‐scale mechanized agroecosystems are one of the most pervasive modes of tropical land cover used by wild large‐bodied vertebrates, such as ungulate across the entire grazer‐browser continuum (Costa et al., 2021).

The white‐lipped peccary (*Tayssu pecari*, VU) is a unique ~30‐kg Neotropical ungulate that forms large herds that can surpass 300 (Emmons & Feer, 1997) individuals which would amount to a herd biomass larger than 12 tons. White‐lipped peccaries (hereafter, WLPs) thus affect forest structure and function through active foraging (rooting, trampling, and feeding) and wallowing and are often considered as ecosystem engineers (Beck et al., 2010; Keuroghlian & Eaton, 2009). WLPs are also wide‐ranging seed predators (Beck, 2006) that exert large impacts on both the quantity and spatial distribution of large‐seeded plants (Silman et al., 2003). Their pivotal ecological role includes reducing rates of seed predation by smaller vertebrate herbivores and bruchine beetles (Coleoptera: Chrysomelidae) (Galetti et al., 2015), limiting the abundance of tree seedlings, and augmenting landscape‐scale plant beta‐diversity (Villar et al., 2020). WLPs also exert a substantial impact on the nutrient cycling of tropical forests (Villar et al., 2021).

As a large group‐living ungulate, WLPs are extremely sensitive to hunting and habitat fragmentation. In Amazonian forests, WLPs exhibit extensive depletion envelopes around local communities induced by overhunting (Parry & Peres, 2015), even in sparsely populated regions (Abrahams et al., 2017). They are typically extirpated from forest patches smaller than ~1000 ha (Benchimol & Peres, 2015), are absent from highly deforested landscapes (<20% forest cover; Zimbres et al., 2017), and are effectively restricted to a few large forest patches within their typically large home ranges (Jorge et al., 2020). Consequently, the species have now been extirpated from 21% of their historical range, are virtually extinct in Mesoamerica and in many parts of Brazil, with Amazonia and the Pantanal wetlands one of their last habitat strongholds (de Faria Oshima et al., 2021). The first studies available on the home range use and movement patterns by WLPs are contentious because they exhibit unpredictable movements (Kiltie & Terborgh, 1983) and were once considered to be migratory (Bodmer, 1990). However, telemetry and GPS tracking studies have shown that, in fact, they hold stable home ranges varying widely in size according to seasonal resource availability, habitat types, level of habitat fragmentation, and matrix quality (Carrillo et al., 2002; Fragoso, 1998; Jácomo et al., 2013; Jorge et al., 2019; Keuroghlian et al., 2004, 2015; Meyer et al., 2019; Moreira‐Ramírez et al., 2019; Reyna‐Hurtado et al., 2009; Richard‐Hansen et al., 2019). WLPs area wide‐ranging “landscape species” (sensu Sanderson et al., 2002), the spatial and ecological requirements of which can be used to guide conservation planning in human‐modified landscapes.

Here we present the first ranging ecology study of five WLPs herds within large‐scale mechanized agricultural landscapes of Mato Grosso within the Legal Brazilian Amazon, which enconpassess Amazonian forests, the drier Cerrado scrub‐savannahs, and transitional vegetation between these biomes. We use resource selection functions at the population level (Johnson, 1980) to investigate how WLPs selects different habitat features throughout the study area both during and outside the cropping season. We predicted that individual WLP herds exhibit a high degree of site fidelity and maintain stable home range configurations throughout the annual cycle. However, we expected high levels of seasonal variation in home range size due to cycles of super‐abundant resources supplemented by soybean and maize crops. We also expected that native vegetation is a preferred macrohabitat, compared to any other agricultural land cover. Furthermore, large habitat remnants are more likely to be selected than small fragments, and herds were expected to exhibit short commuting travel distances from forest remnants to ensure access to food subsidies in croplands while retreating to forest refugia at other times. In addtion, WLPs are expected to select sites near perennial water bodies such as native arborescent palm swamps within riparian forest strip set‐asides within private landholdings, which likely play an essential role in forest connectivity across the study landscapes. Our findings provide important insigths for applied conservation strategies, elucidating movement patterns of a vulnerable and emblematic species persisting in commodity production landscapes in Latin America.

## 2 MATERIALS AND METHODS

### 2.1 Study area

**FIGURE 1:**
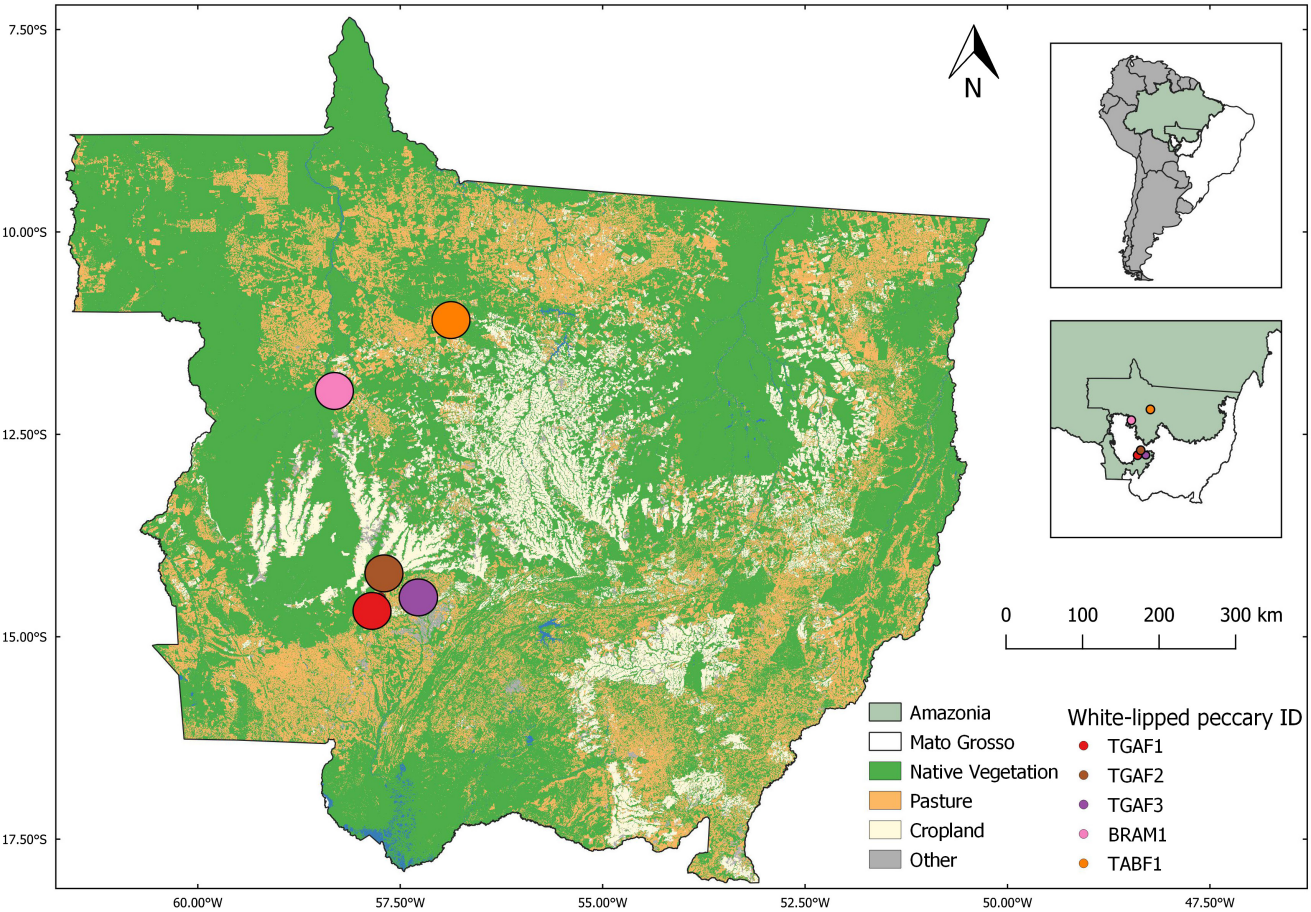
Location of five white‐lipped peccaries (*Tayassu pecari*) to which GPS collars were fitted within large‐scale mechanized agricultural landscapes in Mato Grosso, Legal Brazilian Amazonia.

This study was carried out within the state of Mato Grosso, Legal Brazilian Amazon (−56.736 to −58.359 W and −10.957 to −14.617 S), a region including Amazonian forests, a portion of *Cerrado* scrub‐savannahs and the transitional vegetation between these environments (Marques et al., 2020; Figure 1).

The region has a well‐defined dry and wet season, with annual rainfall and temperature ranging from 15 to 215 mm and from 24 to 36°C. The study landscapes consist of native vegetation remnants embedded within both rain‐fed monoculture croplands and exotic cattle pastures. Croplands consisted of double‐cropping agriculture with soybean usually planted in rotation with maize. The crop season in Mato Grosso extends from mid‐September to mid‐June (wet season), the period that maximizes deterrence of *Phakopsroa* spp. rust, a pathogenic fungi that decimates soybean. Growing soybean is therefore widely banned at other times of year, when all farms become soybean inactive and available for maize intercropping.

### 2.2 WLP capture and handling procedures

Between March and November 2019, we captured five WLP individuals, each of which from independent herds, using live cage traps baited with maize and salt. Traps were placed in the interior of natural habitat remnants surrounded by agricultural land‐use, but within 1000 m from the nearest cropland or pasture edge. Trapped animals were sedated with an intramuscular anesthetic (0.9 mL/10 kg) of Tiletamine Hydrochloride and Zolazepam Hydrochloride (Zoletil50®; Virbac). Handling procedures, following the guidelines from the American Society of Mammalogists (Sikes et al., 2011) and Keuroghlian and Desbiez (2010), were conducted under the SISBIO research permit #60706‐2. All animals were fitted with Telonics TGW4570 GPS collars programed to record relocation data every 2 h, which were relayed to a web server every 2–3 days via two‐way satellite communication (Iridium®). To ensure full recovery from anesthetics, we released the animals 6–12 h after sedation. Subsequent monitoring confirmed that all animals rejoined their herds shortly after release.

Three adult females were captured at Tangará da Serra (TGAF1, TGAF2, and TGAF3), one adult male at Brasnorte (BRAM1) and another adult female at Tabaporã (TABF1). All animals were on average monitored for 367.4 (215–641) days and transmitted 6404 (149–2747) GPS relocations (Table 1; Figure S1).

**TABLE 1:** Animal ID, sex, capture location, number tracking days and number of relocations of five individuals of white‐lipped peccaries (*Tayassu pecari*) equipped with GPS collars at large‐scale mechanized agricultural landscapes in Mato Grosso.

Animal ID	Sex	Capture location	Number of tracking days	Number of GPS fixes		
				Total	Crop	Non crop
TGAF1	Female	Tangará da Serra	641	2747	2021	726
TGAF2	Female	Tangará da Serra	251	1774	1367	407
TGAF3	Female	Tangará da Serra	288	601	541	140
BRAM1	Male	Brasnorte	215	149	149	
TABF1	Female	Tabaporã	442	1053	771	282

TGAF1 was captured on March 2, 2019, and monitored for 641 days until 4th December 2020, when collar battery was drained. We lost the TGAF2 collar signal after 251 days after its capture on July 13, 2019, likely due to GPS antenna damage as the VHF signal was still active. We detected mortality signals from TGAF3 and BRAM1 after 288 and 215 days, respectively. We were unable to pinpoint the cause of mortality due to advanced stage of carcass decomposition when GPS collars were finally recovered. Finally, the collar of TABF1 transmitted data for 442 days after its capture on November 18, 2019.

### 2.3 Landscape predictors

We created seven high‐resolution (30 m × 30 m) landscape and vegetation variables representing habitat structure and composition using land cover maps provided by the MapBiomas collection 5.0 (MapBiomas 2020). First, we estimated individual home ranges (see below) using GPS fixes recorded up to February 2021, which were cropped from a state of Mato Grosso raster file. Next, we reclassified the original data into four major land‐use classes that represent the dominant land use categories within each of the home ranges as following: (1) native vegetation: represented by Amazonian Forest, Cerrado, or transitional vegetation; (2) croplands: represented by soybean plantations, (3) exotic pastures; and (4) other: including mainly perennial and other annual crops. We calculated native vegetation cover and patch area within two different moving‐window radii: (1) 250 m, representing the mean step length between two consecutive GPS relocations within the 2‐h programming interval and (2) 1500 m, representing the daily mean step length of all tracked individuals. We also recorded for each image pixel the Euclidean distance to both native vegetation and perennial water bodies (mostly perennial streams) using the cartographic database of Mato Grosso (itermat.mt.gov.br). All predictors were created using the *raster* package (Hijmans & van Etten, 2012) of R 4.0.3 (R Core Team, 2020). From the set of seven (six numeric and one categorical) predictors, we selected the least correlated below a cutoff value (Pearson correlation |*r*| < .75) using the *select vars* function of the *ENMwizard* R package (Heming et al., 2019). This function computes the pairwise correlation matrix between all variables to search for correlation values above the cutoff value and select variables with the lowest mean absolute correlation. We subsequently retained only four predictors: habitat patch area within the 1500‐m moving window, distance to native vegetation; distance to perennial water bodies, and a categorical variable (land‐cover class).

### 2.4 Data analysis

#### 2.4.1 Home range size

We estimated overall home range size of WLPs during both the cropping and noncropping season based on all monitored individuals using the autocorrelated kernel density estimator (AKDE), which allows more accurate home range estimation for often correlated GPS tracking data in both space and time (Fleming et al., 2015). Using the *ctmm* package and the workflow provided by the authors (Calabrese et al., 2016), we first inspected the empirical semi‐variogram of each animal which is a plot of the semi‐variance in relocations as a function of the time lag among observations (Fleming et al., 2014). This measures the distance between any two relocations and computes the variability of distances among all relocation pairs sharing the same time lag. An animal is considered a range resident if the semi‐variogram displays an asymptote; therefore, it is unlikely that an animal will travel more than its home range diameter during even longer periods (Fleming et al., 2014). Subsequently, we fitted three candidate movement models that describes a home range: (1) the independent identically distributed (IID) process, which features a home range, but not its positional autocorrelation; (2) the Ornstein‐Uhlenbeck process (OU) Brownian motion within a home range (i.e., random, undirected movement), which features the autocorrelated positions described by two parameters: home range crossing time (days) and variance (km^2^); and (3) the Ornstein‐Uhlenbeck motion model with foraging included (OUF), which features both the positional and velocity autocorrelation that are described by three parameters: home range crossing time (days), variance (km^2^), and velocity autocorrelation time. All models were fitted via maximum likelihood and ranked based on their AICc values (Burnham & Anderson, 2002). Once an appropriate continuous‐time model had been selected, we estimated the home range size conditional to that model using the AKDE. We searched for seasonal differences in home range size by subsetting GPS relocations in which fixes from 15th September to 15th June were considered as the cropping season, while data from the rest of the year were defined as the non‐cropping season. For comparisons with previous WLP studies, we also calculated the 95% and 50% annual minimum convex polygon (MCP) and kernel densities home range sizes.

#### 2.4.2 Resource selection functions

To examine resource selection at the population level, we pooled the data from all GPS‐tracked WLPs and fitted generalized linear mixed models (GLMMs) for each season separately. This analysis elucidates which landscape features the average individual selects within the agricultural landscape during both the crop and non‐crop season. To fit these models, we generated a set of pseudo‐absences (available points) within each individual home range to be compared with observed locations. To account for the influence of the number of pseudo‐absences in model fitting (Fieberg et al., 2021) we first fitted models with equal, 5, 10, 50, and 100 available locations for each visited location, until the estimated slope coefficients no longer changed. All data preparation and predictor value extractions for each sampling point was conducted using the *amt* package (Signer et al., 2019) following available code (Fieberg et al., 2021; Muff et al., 2020). For each season, we fitted two different models using the *glmer* function in the *lme4* package, the first one containing a quadratic term for both patch area and distance to forest, a linear term for distance to water‐bodies and land use class as fixed terms. The second model contained only linear relationships for all numeric and categorical predictors. We set the identification of each monitored WLP as a random intercept in the random term of both models. The quadratic relationships for forest patch area and distance to forest were used to examine whether individuals were selecting for intermediate resource values (downward parabola) or both the minimum and maximum available resource values (upward parabola). The linear relationships examined whether WLPs selected the maximum (positive slope) or the minimum resource value (negative slope). We then used the comparative model AICs to examine support for hypotheses regarding the shape of selection (i.e., quadratic vs. linear) for each season separately.

## 3 RESULTS

### 3.1 Overall and seasonal home range sizes

**FIGURE 2:**
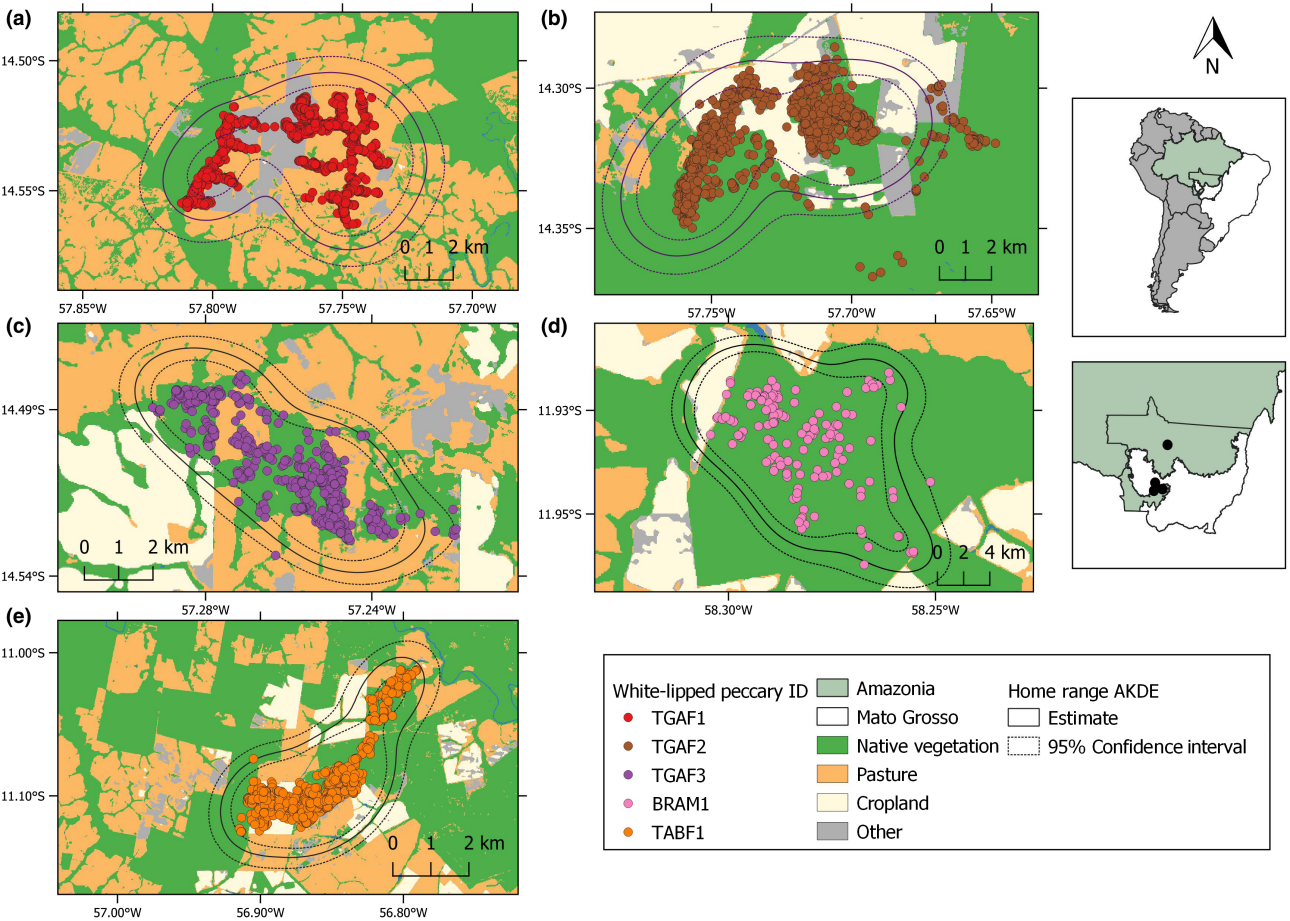
Autocorrelated Kernel Density Estimates (AKDE) of the home range size of five individuals of white‐lipped peccary (*Tayassu pecari*) within large‐scale mechanized agricultural landscapes in Mato Grosso, Brazil. Solid and dotted lines represent the estimated home range size and the 95% confidence interval, respectively.

We were able to estimate overall and seasonal home rage sizes for all WLPs except for BRAM1 which was only monitored during the soybean crop season. Variograms of all individuals displayed an asymptote suggesting that they behaved as strict home range residents (Figure S2). We observed the same pattern when subsetting the data for both the crop and non‐crop season (Figures S3 and S4). The best fit to the data was the Ornstein–Uhlenbeck Foraging (OUF) model revealing overall (Table S1) and seasonal (Table S2) autocorrelation in both geolocations and herd velocity. The largest overall home range size was exhibited by TABF1 (Table 2), followed by TGAF2 and TGAF1. TGAF3 presented the smallest overall home range size within all the monitored females, whereas the adult male BRAM1 exhibited the smallest overall home range size of all tracked (Figure 2; Figure S7).

**TABLE 2:** White lipped peccaries (*Tayassu pecari*) home range sizes (km^2^) at agricultural landscapes in southern Brazilian Amazonia.

Animal ID	95% MCP	95% KDE	95% AKDE		
				Crop season	Non‐crop season
TGAF1	34.29 (7.08)	32.60 (7.75)	43.14 (34.23–53.07)	43.09 (32.48–55.18)	58.24 (36.31–85.26)
TGAF2	39.53 (21.98)	49.97 (10.46)	62.66 (51.13–75.34)	69.21 (54.19–86.05)	62.46 (41.27–87.98)
TGAF3	21.53 (8.73)	28.254 (7.00)	30.91 (21.88–41.47)	32.94 (22.74–44.99)	8.63 (5.03–13.19)
BRAM1	18.33 (4.68)	28.01 (6.58)	26.59 (19.47–34.81)	NA	NA
TABF1	70.03 (9.74)	77.49 (17.25)	125.16 (85.42–172.37)	162.40 (104.59–232.74)	10.11 (7.9–12.59)

*Note*: Minimum convex polygon (MCP), kernel density estimates (KDE) and autocorrelated kernel density estimates (AKDE) were calculated using 95% of GPS collar relocations. Values in brackets represents 50% isopleths for MCP and KDE in km^2^, whereas 95% confidence interval for AKDE estimates.

**FIGURE 3:**
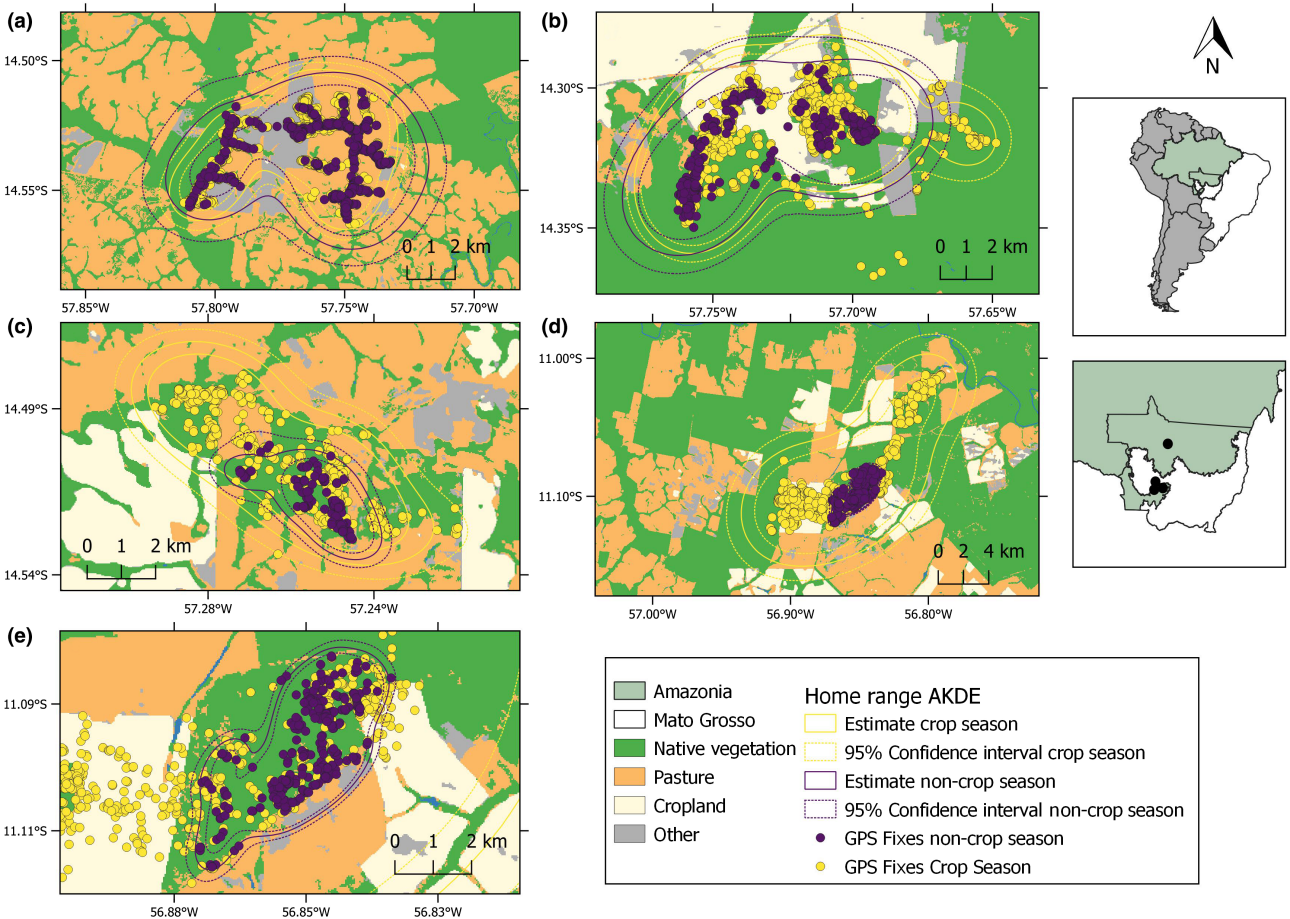
Autocorrelated Kernel Density Estimates (AKDE) of home range sizes of five individuals of white‐lipped peccary (*Tayassu pecari*) during both the crop and non‐crop season within large‐scale mechanized agricultural landscapes in southern Brazilian Amazonia. (a) TGAF1, (b) TGAF2, (c) TGAF3, (d) TABF1 and (e) TABF1. Yellow circles represent GPS relocations during the crop season; purple circles represent the non‐crop season in all figures. Solid and dotted lines represent the estimated home range size and the 95% confidence interval, respectively.

TGAF1 expanded its home range area by 26% between the crop and the non‐crop season (Figure 3a; Table 2). We observed a marked reduction in home range size from the crop to non‐crop season for all other individuals; we did not observe a marked home range reduction in TGAF2 (Figure 3b), whereas TGAF3 used a home range 73% smaller during the non‐crop season (Figure 3c). TABF1 showed the most marked seasonal home range contraction and used a crop season home range 93% larger than during the noncrop season (Figure 3d,e).

### 3.2 Resource selection

Model coefficients hardly changed when models were fitted using 100 available points for each visited location for both seasons and the hypotheses tested (quadratic vs. linear relationships) (Figures S5 and S6). For both seasons, models containing a quadratic relationship with native vegetation patch area and a linear relationship with distance to water bodies were better supported than models that contained only linear relationships (Table 3). The models performed well, with fixed effects explaining considerable variation [*R* ^2^ conditional = .283 (*R* ^2^ marginal = .262) for crop season model; *R* ^2^ conditional = .414 (*R* ^2^ marginal = .257) for non‐crop season model].

**TABLE 3:** Model selection table for white‐lipped peccary resource selection in agricultural landscapes in Southern Amazonia during both crop and non‐crop season.

Season	Fixed terms sturcture	AICc	Delta AICc	Weight
Crop	Patch area^2^ + Forest distance^2^ + Hydrography distance + Land class	130,765.100	0	1
	Patch area + Forest distance + Hydrography distance + Land class	130,940.600	175.420	0
Non‐crop	Patch area^2^ + Forest distance^2^ + Hydrography distance + Land class	42,600.190	0	1
	Patch area + Forest distance + Hydrography distance + Land class	42,634.250	34.051	0

**FIGURE 4:**
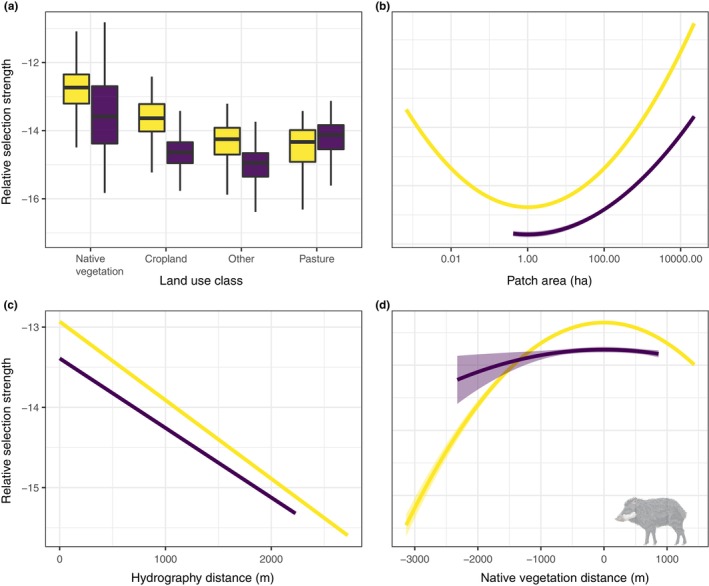
Resource selection of the white‐lipped peccary (*Tayassu pecari*), quantified as the relative selection strength (RSS), within large‐scale mechanized agricultural landscapes in Legal Brazilian Amazonia. RSS in relation to (a) habitat types. Central lines represent the median value, boxes the interquartile range (IQR) and whiskers corresponds to 1.5 times the IQR; (b) patch area; (c) distance to perennial water bodies; and (d) distance to forest patches. Yellow and purple represent the crop and non‐crop season in all figures.

In general, WLPs preferred native vegetation compared to croplands, pastures, and other anthropogenic land use classes regardless of season (Figure 4a; Table 4). However, we observed a marked increase in selection strength for cropland areas and other anthropogenic land use classes during the crop season. Large native vegetation patches were preferred compared to small patches (Figure 4b), with the relative selection strength peaking in fragments larger than 10,000 ha during both the crop (*β* = −.035, *p* = 0) and non‐crop seasons (*β* = −.063, *p* = 0). Locations distant from perennial water bodies were often not at all used by WLPs (crop season, *β* = −.487, *p* = 0; non‐crop season, *β* = −.396, *p* = 0; Figure 4c).

**TABLE 4:** Model coefficient explaining resource selection by white‐lipped peccary in agricultural landscapes in Southern Amazonia during crop and non‐crop season.

Season		Estimate	Std. error	*z*‐Value	*p*‐Value
Crop	Intercept	−12.134	0.171	−70.855	0
	Forest distance^2^	−0.168	0.012	−14.334	0
	Patch area^2^	−0.035	0.007	−5.086	0
	Hydrography distance	−0.487	0.019	−25.168	0
	Cropland	−0.442	0.067	−6.608	0
	Other	−1.957	0.068	−28.701	0
	Pasture	−2.332	0.057	−40.863	0
Non‐crop	Intercept	−12.108	0.505	−23.973	0
	Forest distance^2^	−0.137	0.020	−6.804	0
	Patch area^2^	−0.063	0.012	−5.155	0
	Hydrography distance	−0.396	0.032	−12.531	0
	Cropland	−1.492	0.201	−7.406	0
	Other	−2.758	0.161	−17.173	0
	Pasture	−2.295	0.097	−23.678	0

Although WLPs strongly used native vegetation compared to any other land cover classes and selected large native vegetation remnants, the selection strength for distance to native habitat presented a downward parabola for both the crop (*β* = −.168, *p* = 0) and the non‐crop season (*β* = −.137, *p* = 0). Strength of habitat selection peaked at locations near the boundaries between active cropland and native vegetation (Figure 4d). However, WLPs showed habitat avoidance of distant cropland locations farther than 1000 m from the nearest forest patch and, conversely, farther than 3000 m from cropland edges when using native vegetation. Overall, WLPs showed a higher range of distances commuted to native vegetation during the crop season, compared to the non‐crop season.

## 4 DISCUSSION

This study contributes to our understanding of home range sizes and habitat selection patterns of WLPs in commodity production agricultural landscapes across the Legal Brazilian Amazon, building upon and extending previous research conducted in both protected areas and anthropogenic landscapes. Although these large‐group‐living peccaries represent an iconic Neotropical ungulate, there are inherent difficulties in capturing live‐animals and deploying monitoring collars given the many morphological, behavioral, and environmental constraints associated with this species. For example, a triangular‐shaped head with a very short neck hinders collar fixing, with many devices lost within short periods of data acquisition (Richard‐Hansen et al., 2019). Restrictions in terms of physical accessibility and closed forest canopy environments often preclude adequate positional data collection given typical expectations from device programming (Meyer et al., 2019; Richard‐Hansen et al., 2019). Therefore, spatial ecology studies on WLPs are fairly recent, rarely successful, and concentrated within extensive areas of pristine habitat within formal protected areas. Documentation of the ranging ecology of WLPs is, therefore, almost nonexistent in human‐modified landscapes, where most extant populations now persist.

Although the species has been studied in some agricultural landscapes, all previous studies were conducted exclusively within the scrub‐savannah Cerrado domain. Of these, however, one study was carried out in an area bordering the ~1320‐km^2^ Emas National Park, the largest Cerrado protected area in Brazil (Jácomo et al., 2013). Although another study monitored the species within agricultural landscapes, it failed to distinguish exotic pastures from croplands because of the dominance of the former in the study area (Jorge et al., 2019, 2020). Our study, on the other hand, focuses on properly classified large‐scale double‐cropped export commodity croplands (soybean rotated with maize), representing the most extensive areas of post‐1980 agricultural expansion in Brazil (MapBiomas 2023), the world's leading soybean exporter.

Our estimates corroborate previous studies showing that WLPs are year‐round home range residents regardless of positional data type (VHF or GPS) and statistical approaches to predict home range use (MCP, KDE, or AKDE). WLP home range estimates range widely in size throughout its geographic range according to habitat type, resource availability, and anthropogenic disturbance (Beck et al., 2017, p. 201; Reyna‐Hurtado & Chapman, 2019). The smallest home range (~18 km^2^) was estimated for a small population stranded in a hyper‐fragmented landscape of the Atlantic Forest formed by the 21.8 km^2^ Caetetus Ecological Station, which is embedded within sun coffee plantations and other small forest fragments smaller than 200 ha (Keuroghlian et al., 2004). This estimate is 10‐fold smaller than the ~200‐km^2^ home range size from Manu National Park, Peruvian Amazonia (Kiltie & Terborgh, 1983). Although no tracking devices were used, this home range size is comparable to our largest estimate for agroecosystems in Mato Grosso (125.16 km^2^), the average of 168 km^2^ (*n* = 4 individuals) reported for the Mayan forests of Guatemala and southern Mexico (Moreira‐Ramírez et al., 2019) and the 109.6 km^2^ (*N* = 2 individuals) MCP in the northern Brazilian Amazon (Fragoso, 1998), but smaller than the largest home ranges estimates based on AKDE from hunted sites in Nuevo Becal, Mexico (Moreira‐Ramírez et al., 2019). Meyer et al. (2019) estimated an 100% MCP of only ~8 km^2^ for Darién National Park in Panamá, but these authors monitored WLPs using GPS collars and reported home ranges of 25–59 km^2^ using AKDE. These estimates are similar to those in other tropical and subtropical forest studies using the same approach, such as 49 (32–69) km^2^ and 34 (25–45) km^2^ for a male and a female in French Guiana, respectively (Richard‐Hansen et al., 2019); 55 (34–81) km^2^ in Belize (Hofman et al., 2016), and our average estimates of 57.7 km^2^ from the five individuals we tracked.

Although WLPs often exhibit stable year‐round home ranges, they often wander across larger areas and shift their diet and habitat use in relation to resource availability. This can be seen as they move seasonally, for example, between flooded and unflooded Amazonian forests to take advantage of the higher availability of large seeds settled on the flooded forest floor as floodwaters recede every year (Costa et al., 2018). Similarly, a wet‐season expansion in home range sizes was observed in Cerrado areas of Central Brazil because of super‐abundant resources within soybean and maize available in adjacent croplands (Jácomo et al., 2013). This is also documented in our study, with the exception of TGAF1, the only individual whose home range was embedded within a cattle‐pasture dominated landscape (Table S3), which could not provide high‐quality supplemental food resources during the crop season. WLPs also wandered across areas three‐fold larger at the Calakmul Biosphere Reserve, Mexico, during the wet season, because intermittent ponds – the only water source for wildlife – are no longer critical (Reyna‐Hurtado et al., 2009). At Maracá island, northern Brazilian Amazonia, home range sizes doubled during the wet season when food resources in savannah ponds became abundant and *Mauritia flexuosa* palm clumps were shedding fruits (Fragoso, 1998).

WLPs also travel long distances in landscapes naturally lacking continuous forest cover in response to the sparse distribution of fruiting trees, which may contribute to larger home ranges in human‐fragmented landscapes (Jorge et al., 2019; Keuroghlian et al., 2015). However, this pattern is at odds with our study areas, in which the largest home range estimates were recorded in Tabaporã, where forest cover was most extensive and forest fragments were largest compared to other sites (e.g., Tangará da Serra). Connectivity and landscape configuration contribute to larger spatial requirements in megaherbivores (Walter et al., 2018), although supplemental feeding provided by soybean and maize croplands likely induces more sedentary habitats at least during the maturing crop season. Food availability has been identified as the primary driver in herbivore space use, with both food patch density and food‐quality exerting a negative effect on home range size (van Beest et al., 2011). The animals we monitored within highly fragmented landscapes were often not required to travel long distances since seed croplands adjacent native vegetation remnants and riparian forests could be used as food sources and habitat refugia, respectively, thereby leading to smaller home ranges. WLPs have been described as a migratory species that exploit resources seasonally (Bodmer, 1990) or exhibiting nomadic herd movements within a large, well defined home range (Kiltie & Terborgh, 1983; Peres, 1996). Telemetry studies on WLPs, however, have shown that this species uses stable home ranges and exhibit nomadic behavior within very large home ranges according to regional spatiotemporal variability in resource availability (Carrillo et al., 2002; Fragoso, 1998; Jácomo et al., 2013; Jorge et al., 2019; Keuroghlian et al., 2004, 2015; Meyer et al., 2019; Moreira‐Ramírez et al., 2019; Reyna‐Hurtado et al., 2009; Richard‐Hansen et al., 2019). Large‐scale annual agriculture renders resource availability cyclic and induces epizodic use of space if agroecosystems substantially increase resource periodicity compared to natural landscapes, which likely induces smaller home ranges (Péron et al., 2017). Spatial concentration of herd activity may, however, degrade native habitats and deplete their resources. It is expected, therefore, that individuals leave any given area as soon as it becomes less profitable than the average, returning only when resources have recovered (Bar‐David et al., 2009; Owen‐Smith & Martin, 2015). This generates a periodic pattern of range use related to the expected duration of each depletion/recovery cycle. This cycle is less peaked and much slower in vast tracts of pristine forests, which may partly explain previous interpretations of local extirpations caused by infectious diseases (Fragoso, 1998) and migratory movements (Peres, 1996). Vast expanses of Amazonian upland *terra firme* forests largely lack sufficiently large resource patches that can sustain a large herd of WLPs due to the low heterogeneity of these forests and nutrient‐poor soils. However, seasonal movements may track the spatial distribution of fruiting arborescent palms (Akkawi et al., 2020; Beck, 2006), which are often found in high‐density patches that are considered as keystone plants in sustaining large vertebrates over long periods of food scarcity (Terborgh, 1986).

Our results clearly show that WLPs select native vegetation over any other land cover examined here, and that selection strength highly favors large forest patches. Croplands were the second most used land use type, with higher selection strength during the crop season. When using native vegetation fragments, WLPs preferred locations near the edge of agroecosystems over core forest or Cerrado areas. Accordingly, when using the agricultural matrix, WLPs never moved beyond 1000 m from forest or cerrado fragments. Although WLPs are fragmentation‐sensitive, they can persist in anthropogenic landscapes by either occupying or trap‐lining between large forest fragments (Benchimol & Peres, 2021) or occasionally using matrix areas within a minimum threshold of landscape‐scale forest cover (Zimbres et al., 2017). Employing a multiscale approach, Jorge et al. (2020) demonstrated that white‐lipped peccaries exhibit preferences for forested areas based on their availability at both landscape and home range scales. Notably, their selection for forested habitats was considerably more pronounced at the home range scale. This underscores the significance of forest connectivity for the persistence of white‐lipped peccaries in anthropogenic landscapes characterized by low levels of forest cover. Our analysis corroborates these results, with forest habitat conferring the strongest selection regardless of season of the year, although there was a marked increase in the cropland selection strength during the harvest season. The spatial distribution of food resources largely explains habitat use by WLPs, which can prefer secondary forests over primary forests when resource availability is higher in the former (Altrichter et al., 2001), and use different habitat types within landscape macromosaics throughout the year (Keuroghlian & Eaton, 2009). Although there are no detailed studies on the feeding ecology of WLPs in agricultural landscapes, the species is widely known to exploit seed crops (Magioli et al., 2022), resulting in significant crop raiding conflicts (Lima et al., 2019).

Resources available for WLPs in mechanized agricultural landscapes of Mato Grosso also vary throughout the year, with strong and synchronous pulses of soybean and maize monoculture during the harvest season interspersed with inactive croplands at other times of the year. When supplementary food crops are unavailable, WLPs necessarily shift to wild fruits within native vegetation fragments, thereby markedly shifting their use of space. This explains the upward parabola of forest patch area in relation to selection strength during the crop season, where zero and minimum values represented higher selection for croplands, and maximum values represented large fragments. This was not observed during the non‐crop season, which was characterized by a one‐side upward parabola. Large herbivores usually consume food crops because they are readily available and hyper‐abundant, more nutritious (Sukumar, 1990), contain lower levels of toxins and secondary compounds (Osborn & Hill, 2005), or have low fiber content compared to native plant parts (Hoare, 1999). This seasonally superabundant food supplement also provides ample resources to sustain large herds within relatively small home ranges. However, further studies on the demographic effects of large‐scale intercropping monoculture on WLP population sizes would assess whether they are indeed superabundant and widely regarded as a pest requiring frequent culls as perceived by many farmers in Mato Grosso across the landholding size gradient (Lima et al., 2019).

We have shown that WLP is a large‐group‐living native vegetation specialist herbivore that selects forest and scrub savannah habitats over any other land use type regardless their availability. WLPs can also persist in the long‐term within human‐dominated landscapes, provided that large native vegetation fragments and riparian forests are available. WLPs increase selection for croplands within the harvest season and modulate their home range configuration accordingly to track the spatiotemporal resource periodicity of agricultural landscapes. However, the effects of large‐scale agriculture on and the foraging ecology and populations dynamics of the WLP are still unknown and should be investigated in the future. In addition, effective measures to mitigate crop raiding human‐wildlife conflicts within agricultural landscapes should be considered a high priority by both researchers and decision makers. This could reduce or preclude widespread cases of retaliatory killings by landholders, who often call for WLP eradication rather than population control (Lima et al., 2019), thereby inhibiting the persistence of this species outside protected areas and indigenous territories.

## AUTHOR CONTRIBUTIONS

**Hugo C. M. Costa:** Data curation (equal); formal analysis (equal); funding acquisition (equal); investigation (equal); methodology (equal); writing – original draft (equal). **Danielle Storck‐Tonon:** Conceptualization (equal); methodology (equal); supervision (equal); writing – review and editing (equal). **Manoel dos Santos‐Filho:** Resources (equal); writing – review and editing (equal). **Dionei José da Silva:** Resources (equal); writing – review and editing (equal). **João Vitor Campos‐Silva:** Supervision (equal); writing – review and editing (equal). **Carlos A. Peres:** Conceptualization (equal); funding acquisition (equal); methodology (equal); project administration (equal); supervision (equal); writing – review and editing (equal).

## CONFLICT OF INTEREST STATEMENT

The authors declare no conflict of interest.

## Supporting information


Figure S1.
Figure S2.
Figure S3.
Figure S4.
Figure S5.
Figure S6.

Table S1.
Table S2.
Table S3.

## Data Availability

Data is available at the Movebank repository ID 1208812623.
