# Supplementary material for: Ranging ecology and resource selection of white‐lipped peccaries (Tayassu pecari) in the world's largest tropical agricultural frontier
Source: Ecol Evol. 2023 Oct 18;13(10):e10624. doi: 10.1002/ece3.10624 (PMC10585122; doi:10.1002/ece3.10624)
Supplement: Supplementary file 1 — Figure S1. Figure S2. Figure S3. Figure S4. Figure S5. Figure S6. [file ECE3-13-e10624-s001.docx]

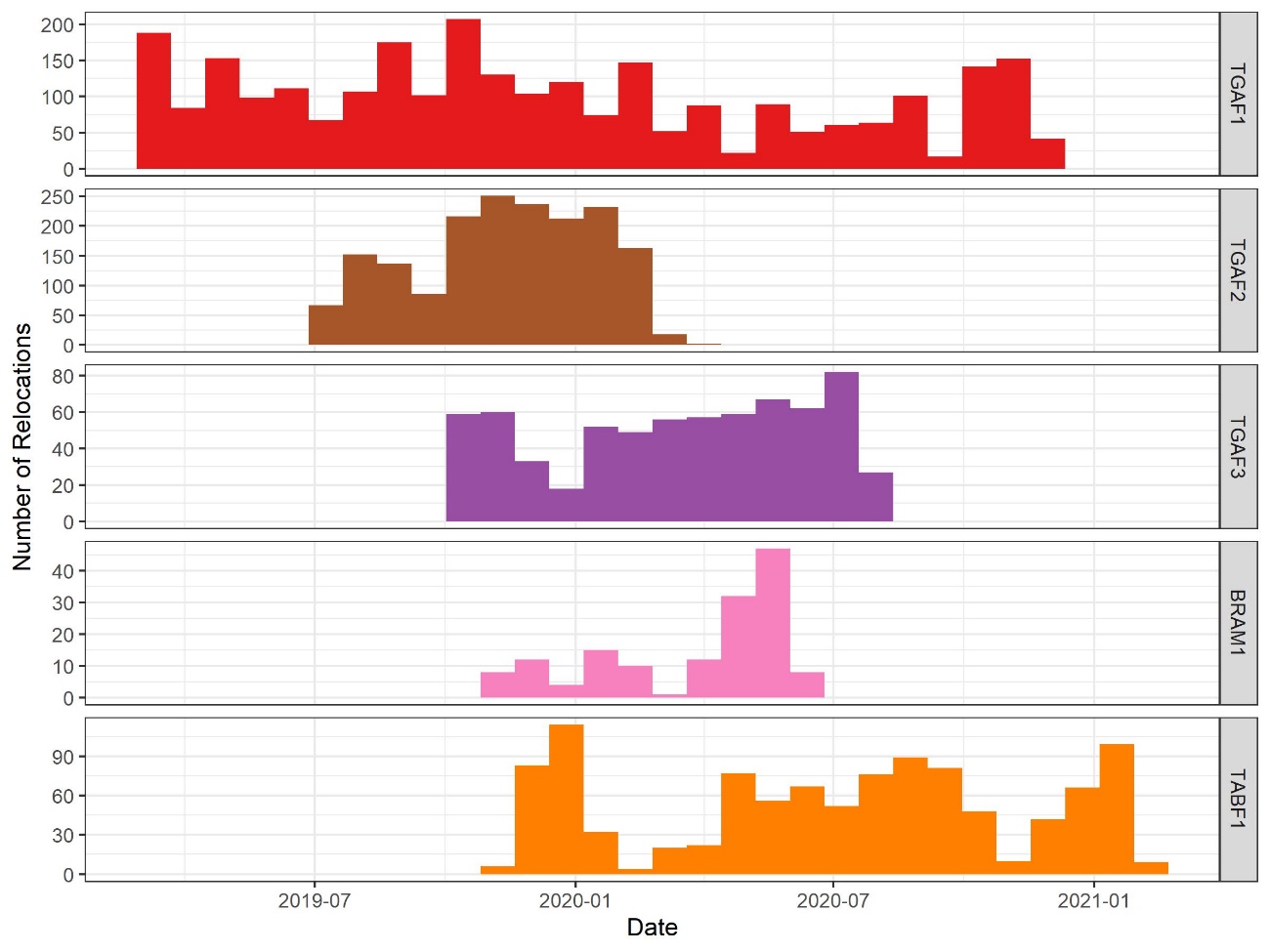


Figure S1. Number of relocations and monitoring period of five individuals of white-lipped peccaries (*Tayassu pecari*) equipped with GPS collars at large-scale mechanized agricultural landscapes in Mato Grosso.


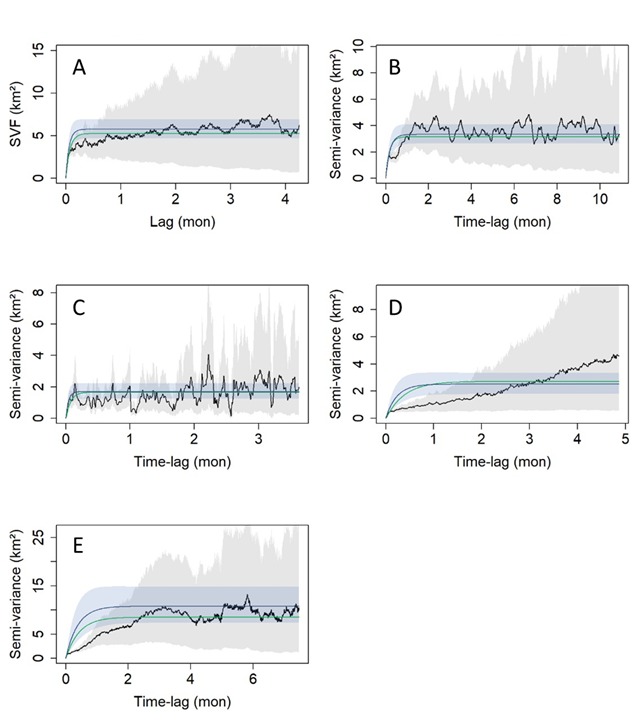


Figure S2. Semi-variograms of the GPS fixes of five resident white-lipped peccaries from different herds (A) TGAF2, B) TGAF1, C) BRAM1, D) TGAF3 and E) TABF1) in agricultural landscapes in southern Brazilian Amazonia. Empiral data is represented in green whereas the Ornstein-Uhlenbeck foraging model was fitted in blue with its 95% CI (blue shading).


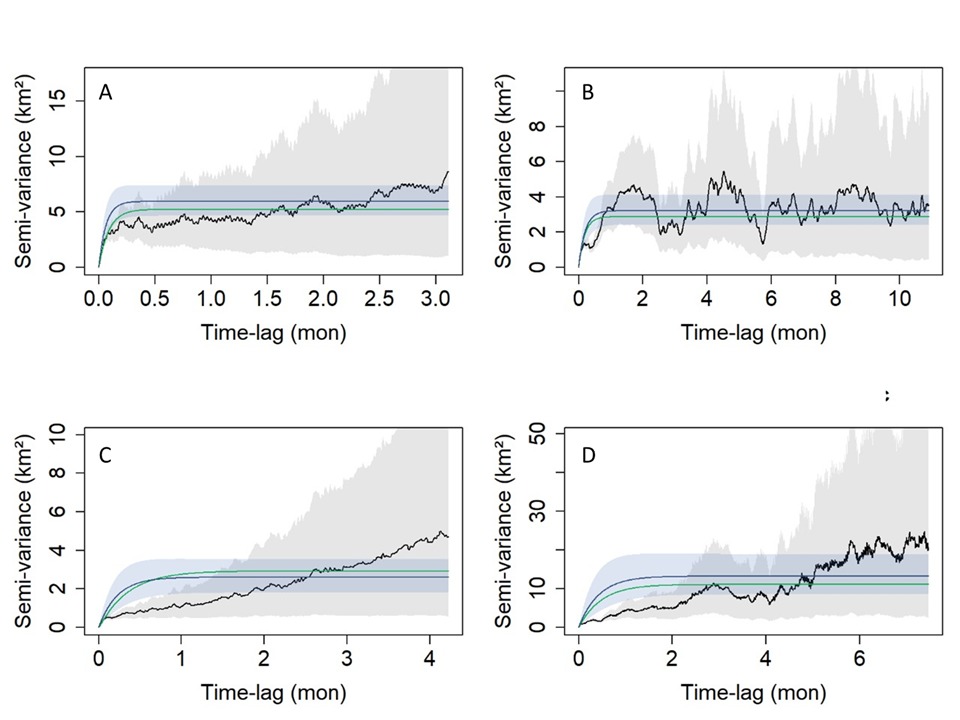


Figure S3. Semi-variograms of GPS fixes during the crop season of four white-lipped peccaries from different herds (A) TGAF2, B) TGAF1, C) TGAF3 and D) TABF1) in agricultural landscapes in Legal Brazilian Amazonia. Empirical data is represented in green whereas the Ornstein-Uhlenbeck foraging model was fitted in blue with its 95% CI (blue shading).


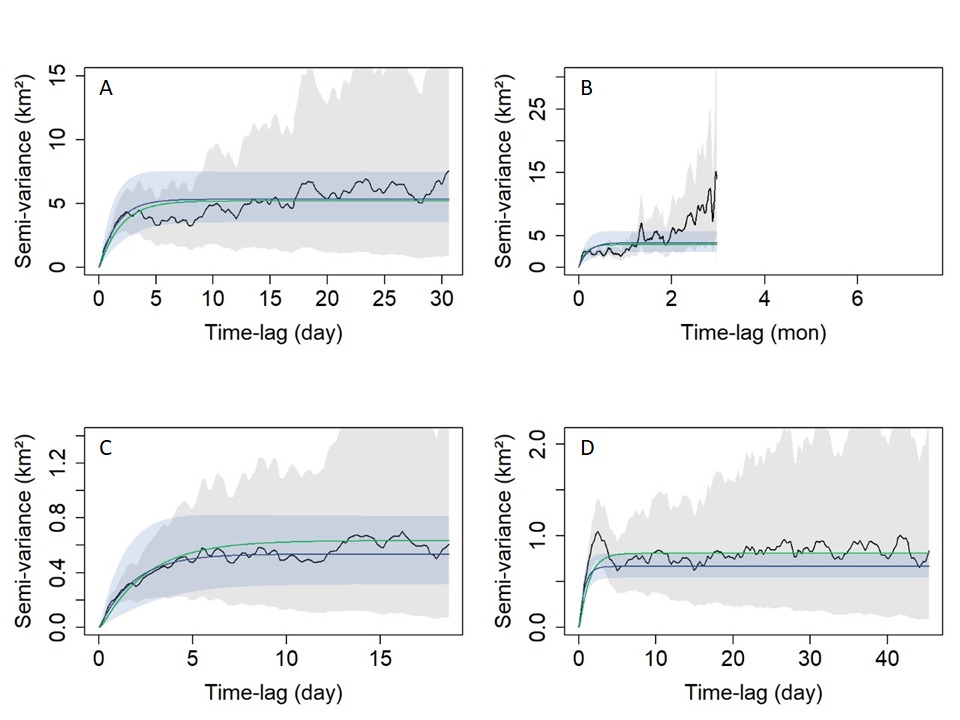


Figure S4 Semi-variograms of GPS fixes from non-crop season of four white-lipped peccaries from different herds (A) TGAF2, B) TGAF1, C) TGAF3 and D) TABF1) in agricultural landscapes in Legal Brazilian Amazonia. Empirical data is represented in green whereas the Ornstein-Uhlenbeck foraging model was fitted in blue with its 95% CI (blue shading).


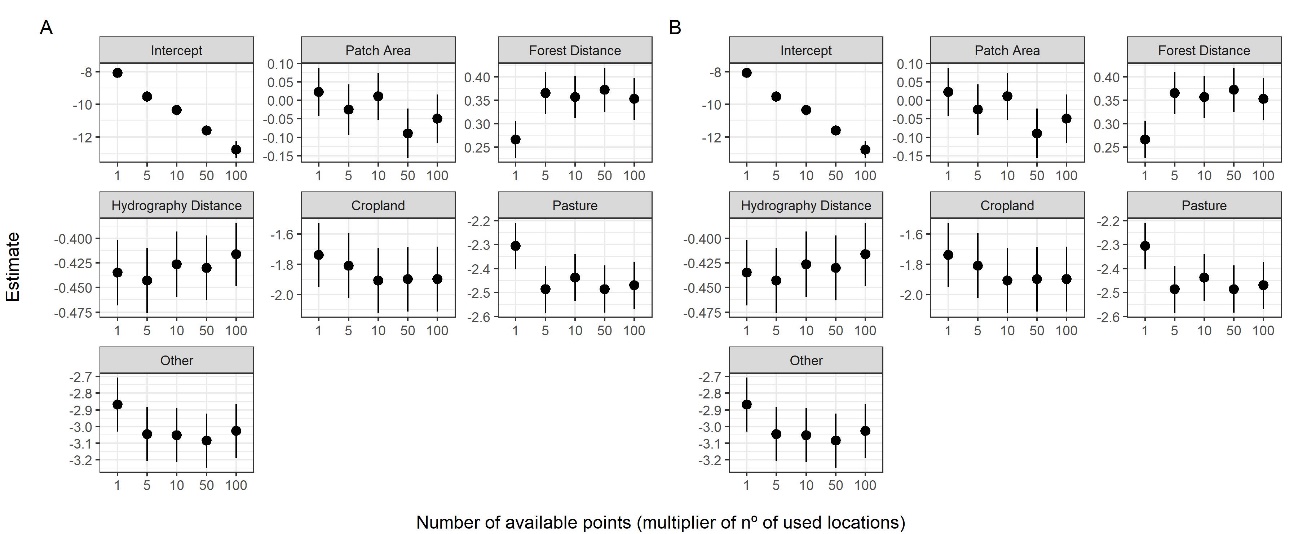


Figure S5. Estimated parameters of resource selection functions fitted using increasing number of random pseudo-absences in relation to presence locations from GPS collars of five individuals of white-lipped peccary (*Tayassu pecari*) during the crop season at agricultural landscapes in Mato Grosso, southern Brazilian Amazonia. A) parameter estimates of resource selection functions containing only linear relationships and B) parameter estimates of resource selection functions fitted with a quadratic relationship for both patch area and forest distance and linear relationship with hydrography distance.


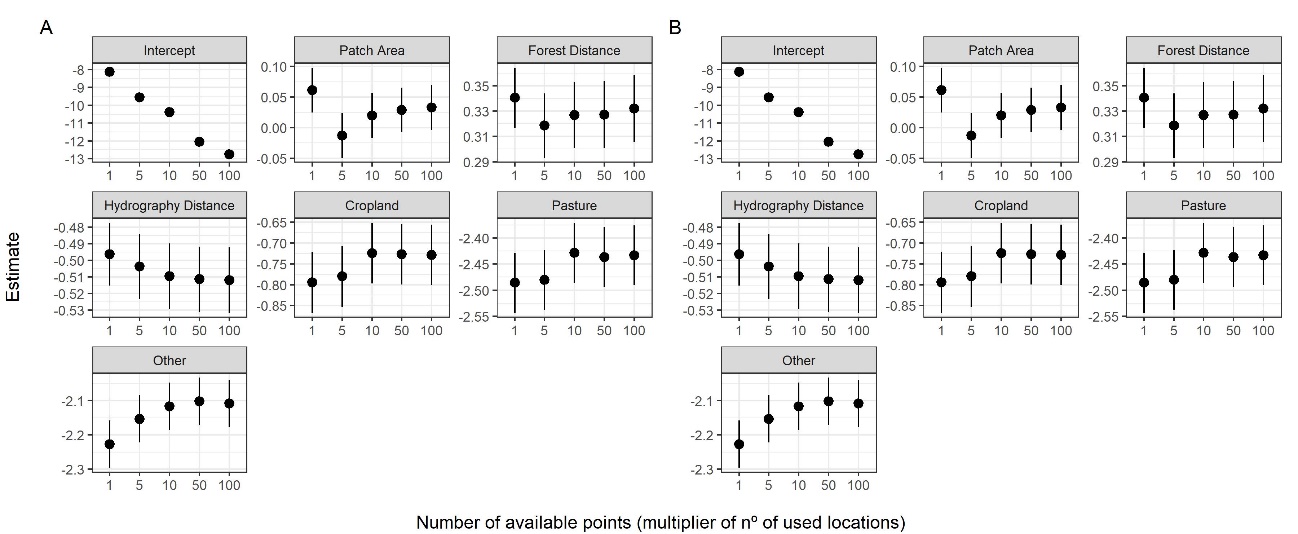


Figure S6. Estimated parameters of resource selection functions fitted using an increasing number of random pseudo-absences in relation to presence locations based on five GPS-tracked individuals of white-lipped peccary (*Tayassu pecari*) during the non-crop season of agricultural landscapes in Mato Grosso. Parameter estimates of resource selection functions containing (A) only linear relationships; and (B) a quadratic relationship for both patch area and forest distance and linear relationship with distance to water bodies.


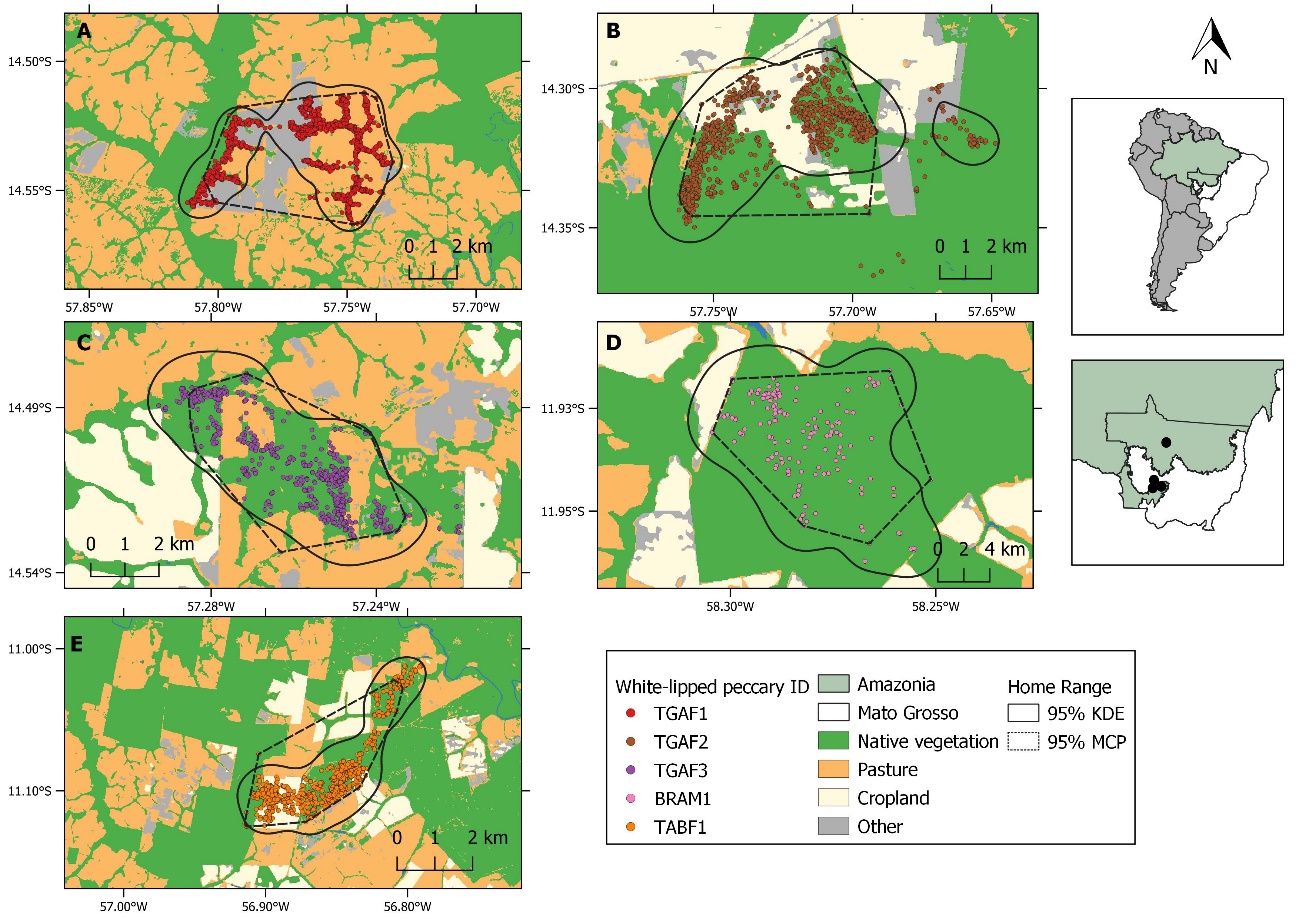


Figure S 7. Kernel Density Estimates (KDE) and Minimum Convex Polygon of home range sizes of five individuals of white-lipped peccary (*Tayassu pecari*) within large-scale mechanized agricultural landscapes in southern Brazilian Amazonia. A) TGAF1, B) TGAF2, C) TGAF3, D) TABF1 and E) TABF1.
