# Supplementary material for: Ranging ecology and resource selection of white‐lipped peccaries (Tayassu pecari) in the world's largest tropical agricultural frontier
Source: Ecol Evol. 2023 Oct 18;13(10):e10624. doi: 10.1002/ece3.10624 (PMC10585122; doi:10.1002/ece3.10624)
Supplement: Supplementary file 2 — Table S1. Table S2. Table S3. [file ECE3-13-e10624-s002.docx]

Table S 1. Model selection table of continuous time movement models fitted to GPS tracked white-lipped peccary (*Tayassu pecari*) individuals at agricultural landscapes in southern Amazonia, Brazil. Best fitted models are highlighted in bold

| Identity | Model type | ∆AICc | DOF mean | DOF area | Area (km²) | Area CI (km²) |
| --- | --- | --- | --- | --- | --- | --- |
| **TGAF1** | **OUF anisotropic** | **0** | **49.76** | **80.39** | **62.44** | **(49.54 - 76.81)** |
| TGAF1 | OUF isotropic | 15.69 | 48.28 | 78.47 | 65 | (51.42 - 80.14) |
| TGAF1 | OU anisotropic | 353.75 | 35.81 | 59.74 | 62.32 | (47.53 - 79.09) |
| TGAF1 | OUf anisotropic | 1846.02 | 293.61 | 557.31 | 41.32 | (37.96 - 44.82) |
| **TGAF2** | **OUF anisotropic** | **0** | **62.53** | **102.75** | **108.09** | **(88.21 - 129.97)** |
| TGAF2 | OUF isotropic | 3.66 | 63.14 | 107.55 | 107.44 | (88.09 - 128.67) |
| TGAF2 | OUf anisotropic | 428.8 | 201.68 | 342.46 | 79.75 | (71.53 - 88.41) |
| TGAF2 | OU anisotropic | 617.27 | 35.82 | 61.23 | 105.79 | (80.96 - 133.88) |
| TGAF3 | OU anisotropic | 35.2 | 18.53 | 30.93 | 45.48 | (30.89 - 62.85) |
| TGAF3 | OUF isotropic | 46.41 | 18.97 | 32.59 | 56.74 | (38.95 - 77.81) |
| TGAF3 | OUf anisotropic | 611.12 | 158.41 | 239.15 | 29.4 | (25.8 - 33.25) |
| **TGAF3** | **OUF anisotropic** | **0** | **22.62** | **38.1** | **45.46** | **(32.19 - 61)** |
| **BRAM1** | **OUF anisotropic** | **0** | **34.14** | **45.98** | **30.96** | **(22.66 - 40.53)** |
| BRAM1 | OUF isotropic | 4.12 | 33.69 | 46.27 | 33.09 | (24.25 - 43.28) |
| BRAM1 | OUf anisotropic | 18.04 | 51.69 | 69.06 | 28.66 | (22.3 - 35.8) |
| BRAM1 | OU anisotropic | 25.32 | 28.08 | 38.53 | 29.84 | (21.17 - 39.98) |
| **TABF1** | **OUF anisotropic** | **0** | **20.01** | **31.66** | **198.3** | (135.34 - 273.1) |
| TABF1 | OU anisotropic | 22.98 | 17.57 | 28.19 | 197.56 | (131.48 - 276.87) |
| TABF1 | OUF isotropic | 42.58 | 17.69 | 28.87 | 230.79 | (154.42 - 322.27) |
| TABF1 | OUf anisotropic | 1237.83 | 196.89 | 319.96 | 105.99 | (94.69 - 117.91) |

Table S 2. Model selection table of continuous time movement models fitted to GPS tracked white-lipped peccary (*Tayassu pecari*) individuals at agricultural landscapes in southern Amazonia, Brazil. Best fitted models are highlighted in bold

| Season | Identity | Model type | ∆AICc | DOF mean | DOF area | Area (km²) | Area CI (km²) |
| --- | --- | --- | --- | --- | --- | --- | --- |
| Non-Crop | **TGAF1** | **OUF anisotropic** | **0** | **14.76** | **21.58** | **72.31** | **(45.08 - 105.86)** |
|  | TGAF1 | OUF isotropic | 10.15 | 14.41 | 21.28 | 74.69 | (46.4 - 109.6) |
|  | TGAF1 | OU anisotropic | 129.83 | 10.34 | 14.61 | 72.21 | (40.06 - 113.68) |
|  | TGAF1 | OUf anisotropic | 415.51 | 73.91 | 134.63 | 46.7 | (39.14 - 54.91) |
|  | **TGAF2** | **OUF anisotropic** | **0** | **18.6** | **27.3** | **98.85** | **(65.31 - 139.23)** |
|  | TGAF2 | OUF isotropic | 8.45 | 17.21 | 26.43 | 111.99 | (73.43 - 158.55) |
|  | TGAF2 | OUf anisotropic | 89.2 | 52.81 | 82.93 | 73.86 | (58.82 - 90.58) |
|  | TGAF2 | OU anisotropic | 124.72 | 11.58 | 17.82 | 95.42 | (56.38 - 144.55) |
|  | **TGAF3** | **OUF anisotropic** | **0** | **11.04** | **17.03** | **9.43** | **(5.5 - 14.41)** |
|  | TGAF3 | OU anisotropic | 11.41 | 8.32 | 12.64 | 9.42 | (4.97 - 15.28) |
|  | TGAF3 | OUF isotropic | 14.08 | 10.06 | 16.08 | 11.44 | (6.55 - 17.67) |
|  | TGAF3 | OUf anisotropic | 47.7 | 35.1 | 53.46 | 7.58 | (5.68 - 9.74) |
|  | **TABF1** | **OUF anisotropic** | **0** | **58.43** | **91.03** | **11.58** | **(9.32 - 14.08)** |
|  | TABF1 | OUf anisotropic | 22.51 | 94.13 | 137.13 | 10.6 | (8.9 - 12.45) |
|  | TABF1 | OUF isotropic | 37.02 | 48.84 | 83.9 | 14.58 | (11.63 - 17.86) |
|  | TABF1 | OU anisotropic | 59.82 | 41.46 | 71.19 | 11.29 | (8.82 - 14.07) |
| Crop | **TGAF1** | **OUF anisotropic** | **0** | **36.05** | **55.19** | **60.16** | **(45.34 - 77.03)** |
|  | TGAF1 | OUF isotropic | 20.71 | 34.64 | 53.23 | 63.46 | (47.57 - 81.61) |
|  | TGAF1 | OU anisotropic | 230.31 | 26.43 | 40.79 | 61.33 | (43.97 - 81.53) |
|  | TGAF1 | OUf anisotropic | 1397.94 | 222.75 | 421.98 | 38.22 | (34.66 - 41.95) |
|  | **TGAF2** | **OUF anisotropic** | **0** | **43.83** | **72.32** | **111.51** | **(87.3 - 138.64)** |
|  | TGAF2 | OUF isotropic | 4.78 | 45.87 | 78.37 | 106.24 | (84.03 - 131.02) |
|  | TGAF2 | OUf anisotropic | 337.96 | 149.09 | 258.49 | 81.1 | (71.51 - 91.28) |
|  | TGAF2 | OU anisotropic | 490.81 | 24.3 | 40.24 | 111.67 | (79.86 - 148.71) |
|  | **TGAF3** | **OUF anisotropic** | **0** | **20.02** | **33.49** | **47.22** | **(32.6 - 64.5)** |
|  | TGAF3 | OU anisotropic | 18.33 | 17.04 | 28.35 | 47.21 | (31.46 - 66.11) |
| Table continued |  |  |  |  |  |  |  |
| Season | Identity | Model type | ∆AICc | DOF mean | DOF area | Area (km²) | Area CI (km²) |
| Crop | TGAF3 | OUf anisotropic | 501.27 | 136.32 | 202.84 | 30.56 | (26.5 - 34.91) |
|  | **TABF1** | **OUF anisotropic** | **0** | **15.6** | **24.5** | **244.37** | **(157.37 - 350.21)** |
|  | TABF1 | OU anisotropic | 9.85 | 14.19 | 22.44 | 242.02 | (152.46 - 351.95) |
|  | TABF1 | OUF isotropic | 22.23 | 14.08 | 22.76 | 279.24 | (176.54 - 405.1) |
|  | TABF1 | OUf anisotropic | 957.04 | 138.61 | 216.89 | 139.84 | (121.85 - 159.06) |

Table S 3. Land class percentage within AKDE home ranges of GPS tracked white-lipped peccary (*Tayassu pecari*) individuals at agricultural landscapes in southern Amazonia, Brazil.

| Identity | Native Vegetation | Croplands | Pasture | Other |
| --- | --- | --- | --- | --- |
| TGAF1 | 16.7% | 0.003 % | 53.84 % | 29.15 % |
| TGAF2 | 63.65% | 28.69 % | 0.57 % | 7.12 % |
| TGAF3 | 63.79 % | 0.66 % | 34.7 % | 0.91 % |
| BRAM1 | 99.75 % | 0.004 % | 0.24 % | 0.02 % |
| TABF1 | 51.08 % | 27.65 % | 20.56% | 0.72 % |
